# Focus groups to explore healthcare professionals’ experiences of care coordination: towards a theoretical framework for the study of care coordination

**DOI:** 10.1186/s12875-014-0177-6

**Published:** 2014-12-24

**Authors:** Sabine Van Houdt, Walter Sermeus, Kris Vanhaecht, Jan De Lepeleire

**Affiliations:** Department of Public Health & Primary Care, Academic Center for General Practice, KULeuven - University of Leuven, Kapucijnenvoer 33 blok J box 7001, 3000 Leuven, Belgium; Department of Public Health & Primary Care, Center for Health Services & Nursing Research, KULeuven - University of Leuven, Kapucijnenvoer 35 blok D box 7001, 3000 Leuven, Belgium; Department of Quality Management, University Hospitals Leuven, Herestraat 49, 3000 Leuven, Belgium

**Keywords:** Care coordination, Theoretical models (MESH), Qualitative research, Healthcare professionals, Experiences

## Abstract

**Background:**

Strategies to improve care coordination between primary and hospital care do not always have the desired results. This is partly due to incomplete understanding of the key concepts of care coordination. An in-depth analysis of existing theoretical frameworks for the study of care coordination identified 14 interrelated key concepts. In another study, these 14 key concepts were further explored in patients’ experiences. Additionally, “patient characteristics” was identified as a new key concept in patients’ experiences and the previously identified key concept “quality of relationship” between healthcare professionals was extended to “quality of relationship” with the patient. Together, these 15 interrelated key concepts resulted in a new theoretical framework. The present study aimed at improving our understanding of the 15 previously identified key concepts and to explore potentially previous unidentified key concepts and the links between these by exploring how healthcare professionals experience care coordination.

**Methods:**

A qualitative design was used. Six focus groups were conducted including primary healthcare professionals involved in the care of patients who had breast cancer surgery at three hospitals in Belgium. Data were analyzed using constant comparative analysis.

**Results:**

All 15 previously identified key concepts of care coordination were further explored in healthcare professionals’ experiences. Links between these 15 concepts were identified, including 9 newly identified links.

The concept “external factors” was linked with all 6 concepts relating to (inter)organizational mechanisms; “task characteristics”, “structure”, “knowledge and information technology”, “administrative operational processes”, “cultural factors” and “need for coordination”. Five of these concepts related to 3 concepts of relational coordination; “roles”, “quality of relationship” and “exchange of information”. The concept of “task characteristics” was only linked with “roles” and “exchange of information”. The concept “patient characteristics” related with the concepts “need for coordination” and “patient outcome”. Outcome was influenced by “roles”, “quality of relationship” and “exchange of information”.

**Conclusions:**

External factors and the (inter)organizational mechanism should enhance “roles” and “quality of relationship” between healthcare professionals and with the patient as well as “exchange of information”, and setting and sharing of common “goals” to improve care coordination and quality of care.

## Background

Patients with complex chronic conditions often require care coordination to ensure the quality of their care [1,2]. However, strategies to improve care coordination do not always have the desired results, partly due to incomplete understanding of the key concepts related to care coordination and the links between these key concepts [3-6].

The lack of clarity on care coordination is a result of the many definitions and theoretical frameworks for the study of care coordination. The landmark study in this domain defines care coordination as “the deliberate organization of patient care activities between two or more participants (including the patient) involved in a patient’s care to facilitate the appropriate delivery of health care services. Organizing care involves the marshalling of personnel and other resources needed to carry out all required patient care activities, and is often managed by the exchange of information among participants responsible for different aspects of care”[4].

A clear theoretical framework for the study of care coordination is lacking. An in-depth analysis of existing theoretical frameworks for the study of care coordination identified 14 key concepts and the possible links between these [7]. These key concepts were then further explored in patients’ experiences about care coordination. Additionally, “patient characteristics” was identified as a new key concept in patients’ experiences and the previous identified key concept “quality of relationship” between healthcare professionals was extended to “quality of relationship” with the patient [Van Houdt et al. Patients’ experiences of care coordination: Towards a theoretical framework for the study of care coordination, submitted for publication].

The present study aimed at improving our understanding of these 15 previously identified key concepts and to explore potentially previous unidentified key concepts and the links between these by exploring how healthcare professionals experience care coordination.

## Methods

### Study design

A qualitative research design was used. Healthcare professionals’ experiences with care coordination were explored through focus groups [8]. Data were analyzed using an adaptive theory approach [9,10]. An adaptive theory approach combines inductive and deductive procedures, meaning data were analyzed to confirm, refute and explore the previous identified theoretical concepts and links, but also to explore new theoretical concepts and links between concepts [9,10].

### Theoretical framework

**Figure 1:**
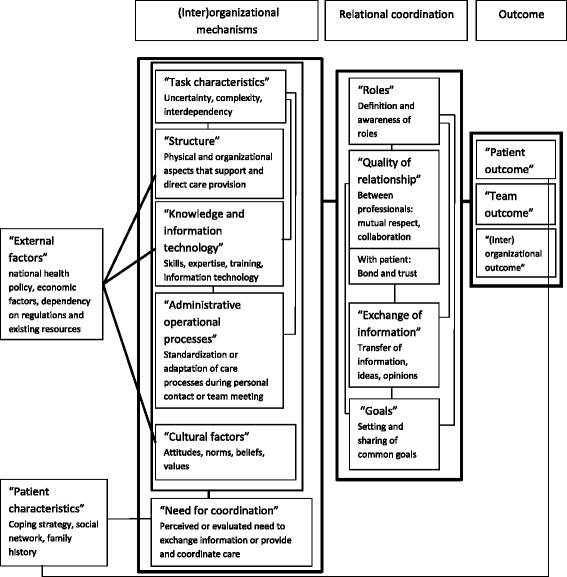
**Interrelated key concepts of care coordination identified within existing theoretical frameworks [** 4 **]** **and patients’ experiences.** [Van Houdt et al. Patients’ experiences of care coordination: Towards a theoretical framework for the study of care coordination, submitted for publication].

The 15 interrelated key concepts identified in the 2 previous studies resulted in a new theoretical framework ([7], Van Houdt et al. Patients’ experiences of care coordination: Towards a theoretical framework for the study of care coordination, submitted for publication). This emerging theoretical framework is presented in Figure 1.

The concept “external factors” linked with three (inter)organizational mechanisms; national health policy, economic factors and dependency on existing regulations and resources (“external factors”) influence (1) physical and organizational aspects that support or direct care, like a structural link across primary and hospital care (“structure”), (2) the availability of (communication) skills, expertise, training and information technology (“knowledge and information technology”) and (3) existing attitudes, norms, beliefs and values of patients and of healthcare professionals towards patients (“cultural factors”).

The fourth identified (inter)organizational mechanism “task characteristics” related to the three other (inter)organizational mechanisms: “structure”, “knowledge and information technology”, and “administrative operational processes”. This means that the characteristics of a task become more complex or uncertain (1) when involved healthcare professionals are not located in one place or when many healthcare professionals are involved (“structure”), (2) when necessary “knowledge and information technology” is lacking and (3) when the care process is not standardized or can’t be adapted during personal contact or team meetings (“administrative operational processes”). “Knowledge and information technology” also linked with “administrative operational processes”. Another (inter)organizational mechanisms, the perceived or evaluated “need for coordination”, was influenced by all the other (inter)organizational mechanisms and “patient characteristics”. “Patient characteristics” refers to coping strategies, social network and family history.

All 6 (inter)organizational mechanisms were linked with the 4 key concepts of relational coordination: (1) definition and awareness of “roles”; (2) “quality of relationship” between healthcare professionals (e.g. mutual respect and collaboration) and with the patient (e.g. bond and trust); (3) timely, frequent, accurate and problem-solving “exchange of information” and (4) the setting and sharing of common “goals”. For example: when the care process is standardized (“administrative operational process”), “roles”, “exchange of information” and common “goals” are defined and shared. Adaptation of the care process is easier when healthcare professionals collaborate and respect each other, hereby increasing the trust of the patient (“quality of relationship”).

The 4 concepts of relational coordination were interrelated. Healthcare professionals who have a good “quality of relationship” know each other’s “role”. If they don’t know each other, they work alongside each other, without knowing who does what. The “role” of a healthcare professional is more extensive if there is a good “quality of relationship” with the patient. Moreover, the role that a healthcare professional performs, can strengthen or decrease the “quality of relationship” with the patient. Healthcare professionals need timely, accurate and problem solving “exchange of information” to perform their “role”. “Exchange of information” is easier when there is a good “quality of relationship” between healthcare professionals. A lack of “exchange of information” is linked with a decrease of the “quality of relationship” with the patient. The setting and sharing of common “goals” support healthcare professionals in their collaboration, in performing their “role” and the accurate “exchange of information”.

Relational coordination resulted in a certain outcome. Three kinds of outcomes were distinguished: patient (e.g. physical or psychological status of the patient), team (e.g. team behavior or team satisfaction), or (inter) organizational outcomes (e.g. comprehensiveness or efficiency of the organization). “Patient outcome” was also influenced by “patient characteristics” (e.g coping strategies of the patient are linked with the psychological wellbeing of the patient).

### Participants

Family doctors, home nurses, and home physiotherapists involved in the care of patients who had breast cancer surgery were invited to participate in a focus group [8]. These primary healthcare professionals were involved in the care of 22 patients who were previously interviewed about how they experienced care coordination [Van Houdt et al. Patients’ experiences of care coordination: Towards a theoretical framework for the study of care coordination, submitted for publication]. All patients consented to contact their primary healthcare professionals. Patients were selected from three hospitals in Belgium in order to achieve cultural diversity. Two focus groups with healthcare professionals were organized in each of these three regions. Since home nurses and home physiotherapists depend on a family doctor’s prescription in order to provide care, healthcare professionals involved in the care of the same patient were invited to different focus groups to stimulate openness.

### Data collection

Focus groups were used for data collection since group interaction stimulates discussions that further explore experiences and views about care coordination. Participants were asked to openly describe their experiences and vision about care coordination. The focus groups were led by an independent moderator using a semi-structured guideline that included the following topics:

1. understanding care coordination;
2. role of each discipline in the care of the patient;
3. monitoring the quality of care;
4. care coordination at a regional level;
5. care coordination at a patient level; and
6. role of the patient.

An additional researcher was present to provide (logistic) support and to note non-verbal communication. Interviews were digitally recorded and transcribed verbatim.

### Data analysis

An adaptive theory approach was used to help to both organize the data and stimulate the process of theoretical thinking [9,10].

Transcripts of the interviews were first re-read as a whole (SVH and JDL) while listening to the recorded interviews (SVH). Next, two researchers (SVH and JDL) assigned and discussed codes to ensure that all relevant codes were identified. During the coding process, both “open” codes and codes derived from the existing theoretical concepts were used. Saturation was reached, meaning that no new codes were identified.

A constant comparative analysis was used to identify concepts emerging on a more abstract level. The final step was to look for a core concept and for links between concepts. A characteristic of a core concept is centrality, meaning that many other concepts are linked to it. Links were identified by determining which concepts facilitated, impeded, influenced, or were related to another concept [8]. The process of constant comparative analysis, and theoretical thinking was guided by our research question and influenced by the theoretical concepts and ideas found in a literature review [8,10]. Nvivo 10 was used to assist with coding, sorting, and retrieval of the data. The fragments were originally recorded in Dutch and then translated into English for inclusion in this paper.

### Ethical considerations

This study was approved by the Ethical Committee of the KU Leuven, University of Leuven. Patient confidentiality and anonymity were guaranteed.

## Results

Twenty of 58 invited (34%) healthcare professionals participated in the focus groups, which included 5 of 24 (21%) general practitioners, 9 of 20 (45%) home nurses, and 6 of 14 (43%) physiotherapists. All participating physiotherapists had a specialization in lymphatic drainage (Table 1). Seventeen of the participating healthcare professionals were female (85%). Participating healthcare professionals had between 1 and 160 patients who had surgery after a diagnosis of breast cancer in the past year, with a median of 4 to 5 patients. Eight healthcare professionals described a special experience with breast cancer, meaning that they had worked at an oncology department in the past (N = 2), treated many breast cancer patients in the Netherlands (N = 2), had had breast cancer themselves in the past (N = 2), were the chairman of the league for cancer (N = 1), or were involved in the development of a care pathway for patients who had surgery after a diagnosis of breast cancer, and had completed training for palliative care (N = 1).

**Table 1:** **Participant focus groups**

	**GP**	**Home nurse**	**Physiotherapist**	**Total**
**Focus group 1**				
**Invited**	4	4	2	10
**Confirmed**	2	3	1	6
**Participated**	0	1	1	2
**Focus group 2**				
**Invited**	4	3	1	8
**Confirmed**	2	2	1	5
**Participated**	0	2	1	3
**Focus group 3**				
**Invited**	4	3	2	9
**Confirmed**	1	2	1	4
**Participated**	1	2	1	4
**Focus group 4**				
**Invited**	4	3	3	10
**Confirmed**	2	2	1	5
**Participated**	1	0	1	2
**Focus group 5**				
**Invited**	4	3	3	10
**Confirmed**	3	2	1	6
**Participated**	2	1	1	4
**Focus group 6**				
**Invited**	4	4	3	11
**Confirmed**	2	5	2	9
**Participated**	1	3	1	5
**Total number invited**	24	20	14	58
**Total number of participants**	5	9	6	20

**Figure 2:**
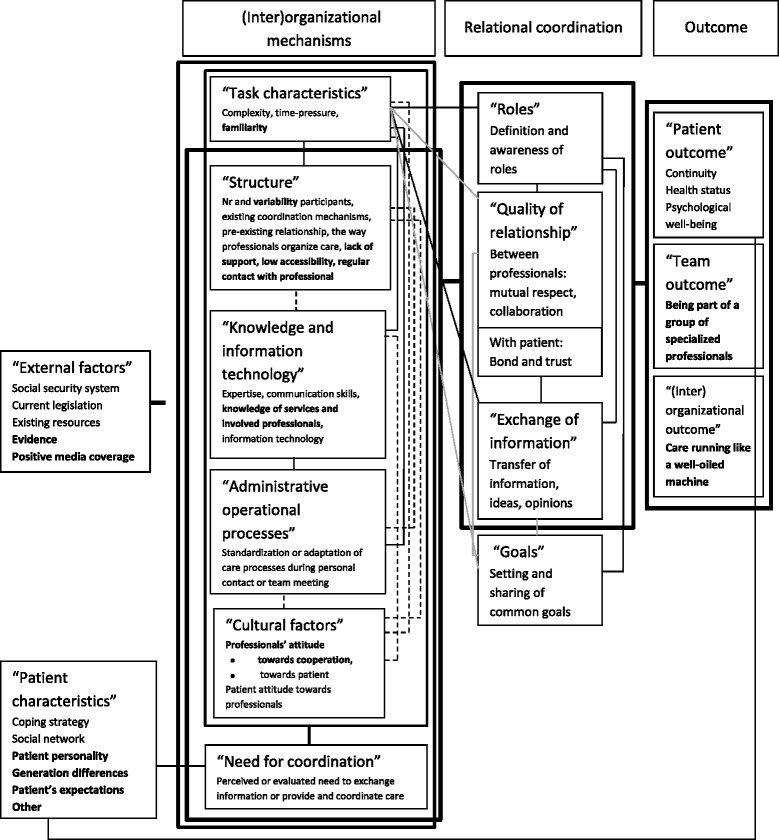
**Key concepts identified in primary healthcare professionals’ experiences of care coordination.** Dashed lines present newly identified links. Bold aspects were newly revealed. Grey lines indicate links not found in primary healthcare professionals’ experiences about care coordination.

The results relating to how healthcare professionals experienced care coordination are illustrated with anonymous fragments from the transcripts. The results are presented in Figure 2.

### Experiences of care coordination

All 15 previously identified key concepts were found and further explored in healthcare professionals’ experiences of care coordination. Additionally, six new links between key concepts relating to (inter)organizational mechanisms and three new links with “external factors” were identified. The results below focus mainly on the newly identified information that enhanced our understanding of key concepts of care coordination and the newly revealed links.

#### External factors

The social security system, current legislation, and existing resources were confirmed to be external factors in healthcare professionals’ experiences about care coordination. Two external factors were newly identified:

1. physiotherapists in three focus groups 1, 2, and 5 mentioned (the lack of) evidence about lymphatic drainage;

*There still isn’t evidence for oedema, but I believe in it. If you remove the lymphatic nodes under the armpit and you don’t begin to drain this from the start, the fluid has to find its way. If you start to show the way for the fluid to drain from the beginning, then channels are built. There is fluid. I really believe in it. (Focus group 1, region 1, participant 2, physiotherapist).*

2) Participants in focus group 6 mentioned the current positive media coverage including several famous people who have survived (breast) cancer.

*My mother had breast cancer many years ago. She told no one because in those days you were doomed if you had breast cancer. Now, breast cancer gets a lot of media coverage, with some famous people having had breast cancer, leading to better treatment adherence (Focus group 6, region 3, participant 16, home nurse).*

#### Patient characteristics

Coping strategies and social network were confirmed as patient characteristics. Four new types of patient characteristics were identified:

1. patients’ personality traits like assertiveness versus resignation, open versus closed, control versus doubt, stubbornness versus docility;

*Some patients talk about it, others don’t. Some patients want to see the wound, others don’t. I know a lady who didn’t want to look in a mirror. (Focus group 6, region 3, participant 16, home nurse).*

2) generational differences like different familial relationships, openness to discuss issues, access to information sources;

*I think there is a difference between older women who have breast cancer and younger ones. We are familiar with the internet, etc. People aged over 70 years who have breast cancer are not familiar with this, and they have a totally different mentality concerning family. I think we should keep a closer eye on these people. (Focus group 6, region 3, participant 16, home nurse)*.

3) expectations of patients towards healthcare professionals or the care provided;

*Patients expect a lot of knowledge. It is not always so obvious to answer their questions. In primary care, we have a lot of different patients; patients with psychiatric problems, patients with heart diseases, etc. We see a lot. It is not easy to know everything (Focus group 1, region 1, participant 1, home nurse).*

4) Other characteristics such as knowledge, education, and motivation.

“Generational differences” were not stated in region 2. “Other” patient characteristics were mentioned by only one participant.

#### (Inter)organizational mechanisms

All six previously identified (inter)organizational mechanisms were found and further explored in healthcare professionals’ experiences.

##### Task characteristics

The complexity of the task and time-pressure were confirmed as task characteristics. The familiarity of healthcare professionals with the task was revealed as a new task characteristic. Primary healthcare professionals are confronted with many different conditions, whereas hospital healthcare professionals specialize in only one. Consequently, primary healthcare professionals had fewer patients with breast cancer and were less familiar with the tasks they had to perform.

*It is difficult to gain experience and know what you have to do if you only have one patient per year. (Focus group 4, region 2, participant 11, family doctor).*

##### Structure

Four physical and organizational aspects that support and direct care were confirmed in healthcare professionals’ experiences; the high number of participants, existing mechanisms for coordinating care, the pre-existing relationship with the patient, and the way healthcare professionals organize work. Also, four new structural aspects were identified:

- the high variability of involved primary and hospital healthcare professionals;

*I think it is very important that you work with a more or less fixed team. Now, you say [to the patient], “find a healthcare professional, do whatever…” (Focus group 1, region 1, participant 1, home nurse).*

- the lack of administrative and logistic support in primary care;

*In primary care, we need more structural support for our administration. (Focus group 4, region 2, participant 11, family doctor)*.

- the poor accessibility of hospital doctors;

*You really should call the hospital and try to contact the oncologist… So our patients call us, and we try to solve the problem. If we can’t, then I sometimes call the hospital. (Focus group 4, region 2, participant 11, family doctor)*.

- and; the regular contacts of the patients with the hospital, home nurses and physiotherapists.

*Healthcare professionals who frequently visit the patient; know the patient; see how the patient lives, speaks, and what questions she has; talk to the patient during care; teach the patient how to deal with the pain and mutilation and ease the patient. That’s essential and irreplaceable. (Focus group 6, region 3, participant 19, family doctor)*.

##### Knowledge and information technology

Participants confirmed two previously identified factors related to knowledge; expertise and experience to undertake the procedures for breast cancer treatment, and communication skills towards the patient.

Two new aspects of knowledge were revealed:

- knowledge about the services and expertise of others;

*If patients call, they almost never ask if I can perform lymphatic drainage, so I suppose that there are patients who end up with physiotherapists without this expertise. (Focus group 6, region 3, participant 18, physiotherapist)*.

- and which primary or hospital caregivers were involved.

*We need information about where the patient is and who is involved with the patient. (Focus group 5, region 3, participant 15, family doctor).*

The family doctors confirmed the importance of the support of information technology. In one region, some family doctors could consult the patient’s hospital record.

*I usually consult the patient’s hospital file. I can log in into the computer of the hospital to determine which patients are hospitalized and which patients are discharged. […]. It is necessary if you want information about certain tests on time, but it is time-consuming. (Focus group 5, region 3, participant 15, family doctor)*.

One physiotherapist had a good experience with a shared electronic patient file for healthcare professionals and insurers in the Netherlands.

##### Administrative operational processes

Standardization and adaptation of the care process were confirmed as important aspects of administrative operational processes. According to the participants, the care process was mainly adapted by phone. Multidisciplinary meetings were considered important, but were not often organized, except in certain local communities.

##### Cultural factors

The attitude of the healthcare professionals towards patients and the attitude of the patients were confirmed as “cultural factors”. The willingness to cooperate was revealed as a new cultural factor, relating to the attitude of healthcare professionals towards other healthcare professionals. Examples of healthcare professionals not willing to cooperate were mainly related to hospital doctors who were perceived as believing that they were superior, focusing only on their own specialty without attention to other aspects, not recognizing the expertise of other healthcare professionals, and acting annoyed when other healthcare professionals asked questions.

*I personally believe that they sometimes have blinders. It is like “I’m only performing this operation and that is for the after-care” (Focus group 2, region 1, participant 4, home nurse)*.

##### Need for coordination

“Need for coordination” was confirmed. No new information about the need for coordination was identified.

#### Relational coordination

All four previously identified concepts of relational coordination were found in healthcare professionals’ experiences: (1) the definition and awareness of “roles”; (2) “quality of relationship” between healthcare professionals and with the patient; (3) timely, accurate and problem-solving “exchange of information”; and (4) setting of common “goals”. “Roles”, “quality of relationship”, and “exchange of information” were identified as core key concepts of care coordination, since they appeared frequently in the data and were linked with most key concepts. The concept “goals” was identified in only one region.

Participants of the focus groups indicated that they experienced a gap in bridging primary and hospital care and in coordinating the primary care. It was unclear who performed this role: the hospital doctor, the specialist nurse, the family doctor, the patient, an engaged healthcare professional, a social assistant or no one. The participants experienced low “quality of relationship” between healthcare professionals, although participants in two regions provided examples of good collaboration with mutual respect. Bond and trust were confirmed as two important aspects of the “quality of relationship” with the patient. All participants stressed that there was a lack of exchange of information, even communicating information towards professionals was deficient.

*In my experience, there is no direct information exchange. As a healthcare professional, you have to pick up the phone and call. (Focus group 3, region 2, participant 6, home nurse)*.

Only participants in region 1 mentioned the setting of common “goals”. Participants in focus group 1 noted that healthcare professionals formulated their own goals together with the patient. They indicated that general goals should be formulated by the family doctor starting from the treatment protocols in hospital. Participants in focus group 2 stated that it was difficult to formulate goals due to the uncertainty of the care process and the disappointments of the patients when goals were not reached. Sharing of goals with other healthcare professionals or the patient was not mentioned.

*You formulate goals, but when they are achieved also depends on the patient […] The more you formulate goals, the more disappointment you will encounter if they have not reached the goal (focus group 2, region 1, participant 5, physiotherapist).*

#### Outcome

Patient, team and (inter)organizational outcomes were confirmed and further explored. Continuity of care, the health status and psychological wellbeing of patients were identified as patient outcomes. Being part of a group of specialized healthcare professionals was considered a positive team outcome. However, most healthcare professionals were not involved in such a group, meaning that the healthcare professionals mainly acted independently of each other with little involvement between them, and little knowledge of who did what or what could be expected, with the possible consequence of looking unprofessional and creating conflict between healthcare professionals.

*Every doctor has his own habits and procedures. There are doctors that you can call between 11 am and 12 am but not after 12 am. If you don’t know the doctor and you call after 12 am, you’re screwed. Next time you call this doctor, it is painful. Maybe the doctor didn’t know that I wasn’t aware of the fact that I couldn’t call after 12 am. This leads to conflicts. If you have a group of people in this region who are specialized and work together, then it runs smoothly. (Focus group 1, region 1, participant 1, home nurse)*.

Care running ‘like a well-oiled machine’ was identified as (inter)organizational outcome, meaning all steps in the care process were done by different healthcare professionals (in different organizations and settings), and follow each other quickly and smoothly (no long waiting periods).

*A first step is that we quickly refer to the hospital when we find something suspicious. In my practice, a mammo often happens in the periphery. Quality of care means that you don’t have to wait too long when there is a suspicious mammo. (Focus group 5, region 3, participant 14, family doctor)*.

#### Identified links between key concepts

##### Links with other concepts

The previously identified link between “patient characteristics” and “need for coordination” was also found in healthcare professionals’ experiences of care coordination.

*It also varies from patient to patient. There are a lot of differences between people. There are people who resolutely take control, and there are people who have a naturally doubtful personality. It always varies from person to person. (Focus group 6, region 3, participant 16, home nurse)*.

The previously identified links between “external factors” and three (inter)organizational mechanisms were found and new links between “external factors” and “task characteristics”, “administrative operational processes”, and “need for coordination” were identified. For example: the social security system, current legislation, and existing resources (“external factors”) were related to the experienced time-pressure of the healthcare professionals (“task characteristics”), no or few multidisciplinary team meetings to adjust care (“administrative operational processes”) and the experienced “need for coordination”.

*The big problem in primary care is that sometimes, I experienced it myself, you are too busy. If you want to care for chronically ill patients well and give them attention, then you have to make time. If you don’t, you will lose certain aspects, and you have to drop things. You need more resources to do it well; like for diabetic patients where we have someone who arranges practical things […] I would like to have more time to contact the home nurse to exchange information and arrange care. But, I can’t call everyone. If I could, I would ask a secretary to call the physiotherapist, home nurse, and home help to arrange a meeting to organize care at home. (Focus group 4, region 2, participant 11, family doctor).*

##### Links between (inter)organizational mechanisms

All six (inter)organizational mechanisms were linked with each other, meaning that all 9 previously identified links were confirmed and six new links were revealed. A link was identified between “cultural factors” on the one hand and “task characteristics”, “structure”, “knowledge and information technology”, and “administrative operational processes” on the other hand, and also between “structure” on the one hand and “knowledge and information technology” and “administrative operational processes” on the other hand. For example, the willingness of healthcare professionals to cooperate or their commitment to the care of a patient (“cultural factors”) is linked to their workload (“task characteristics”), the way they organize their work (“structure”), gaining “knowledge”, using available “information technology”, and contacting another healthcare professional to adjust care (“administrative operational processes).

*You have to discuss everything with the others. If there is something that you don’t know, you have to refer the patient or gain information from someone who does know. You have to know your own boundaries. I don’t have any problems giving a patient a note to ask if that’s normal. (Focus group 1, region 1, participant 2, physiotherapist).*

##### Links between concepts of relational coordination

The previously identified links between “roles” on the one hand and “quality of relationship” with the healthcare professionals and with the patient, “exchange of information” and “goals” on the other hand were confirmed. Also the previously identified links between “exchange of information” on the one hand and “quality of relationship” on the other hand were identified.

*It is unprofessional if you arrive at a patient’s home and you don’t know the medical history and you don’t know anything. It doesn’t give much confidence to the patient. (Focus group 3, region 2, participant 6, home nurse).*

##### Links between (inter)organizational mechanisms and concepts of relational coordination

All previously identified links between (inter)organizational mechanisms and “roles”, “quality of relationship”, and “exchange of information” were found except for “task characteristics” for which we only found a link with “roles” and “exchange of information”.

*I have half an hour per patient. I think home nurses have less time. During this time, the patient talks and knows that she can ask questions. If there is something I don’t know I refer them to the home nurse or the family doctor. Once they know you from the start, they know they can ask you questions. (Focus group 2, region 1, participant 5, physiotherapist)*.

##### Links with outcome

Outcome was related to “roles” healthcare professionals perform, the “quality of relationship” between healthcare professionals and with the patient and “exchange of information”. Participants also indicated that “patient characteristics”, like patient personality, influenced “patient outcome”.

*Why do things go wrong? If patients come too late, if patients mention problems too late… (Focus group 2, region 1, participant 5, physiotherapist)*.

## Discussion

All 15 previously identified key concepts were found and further explored in healthcare professionals’ experiences of care coordination, resulting in more well-grounded key concepts of care coordination. Also, links between the key concepts of care coordination were identified including six new links between key concepts related to (inter)organizational mechanisms, and three new links to “external factors”. “External factors” were related to all interrelated (inter)organizational mechanisms. These mechanisms were linked with “role”, “exchange of information”, and all except “task characteristics” with “quality of relationship”. There was a link between these three concepts of relational coordination and outcome. “Patient characteristics” influenced the “need for coordination” and “patient outcome”. These results are presented in Figure 2.

Strengths of this study are that the results were confirmed in three different regions. Saturation was reached, assuring that all theoretical concepts were retrieved. Since we started with a phenomenological approach to data collection, we aimed at small groups with one participant from each discipline to gain in-depth insight into the experiences of primary healthcare professionals. We succeeded in forming focus groups with a limited number of participants. Despite several attempts, e.g. like contacting healthcare professionals by phone, and changing the dates of three focus groups at the request of the participants, only three focus groups had representatives from all disciplines present. The main raison for not participating was consultation time. Another limitation is that we focused on care coordination in primary care and the transition between primary and hospital care for patients who had breast cancer surgery. Participants noted that hospital care is rather well coordinated for patients who had breast cancer surgery in comparison with other patient conditions, but they experienced a gap between the primary and hospital care. Occasionally, participants gave examples of other patient conditions. Nevertheless, the results are likely to apply to other patient conditions since our focus was not on the condition, but on the process of care coordination across boundaries of disciplines, organizations, and settings. Finally, all steps in this study were discussed with minimum two researchers to exclude possible bias. One of the researchers was both researcher and clinician. This researcher was involved in the analysis and the interpretation of the data. Analysis and interpretation was discussed with a second researcher. All disagreements were resolved. Results were also presented to and confirmed by the other researchers and several hospital and primary healthcare professionals.

Eight of the key concepts identified in this study were also found in a study on important components of cancer care coordination, thereby supporting our findings: 1) support to organize and navigate through care, a liaison between different providers and settings, the distance to the healthcare professionals, the accessibility and having one key contact corresponded with our key concept “structure”; 2) the “role” of the healthcare professional to refer, advise, support, and inform patients, and a strong focus on assessing and evaluating patients physical, psychological, and supportive needs during the cancer care journey to help empower patients in managing the challenges of their disease; 3) “exchange of information” between a multidisciplinary team and other health service providers; 4) “quality of relationship” between the patient and key contact and cooperation between team members; 5) the perceived “need” of the patient; 6) depending on “patient characteristics”; 7) providing confidence, lowering anxiety, providing the perception of seamless care, and sufficient, timely information in a way such that patients understand the particular aspects of their care plan as “patient outcome”; and 8) to deliver care in a complementary and timely manner as an “(inter)organizational outcome” [11].

Participants experienced a lack of care coordination in primary care and across the primary-hospital care continuum. It was unclear who currently coordinates primary care at a patient level in Belgium. Several stakeholders were mentioned, including patients. Healthcare professionals indicated that patients have an important “role” in the management of their own care. Patients therefore need all relevant information, given to them in a way that they can understand, and require specific guidance, key background knowledge, and certain skills [12]. They also need a healthcare professional with whom they have a good “quality of relationship”. Some patients have this relationship with their family doctor. Whether a patient has a personal family doctor was associated with their perception of its importance and with factors that create an opportunity for a relationship to evolve. This personal family doctor-patient relationship becomes more important in case of more serious or psychological problems [13].

Although setting and sharing common goals is considered important, our results show that it rarely happens in daily practice. A study of the experiences of patients with long term conditions relating to care planning confirmed that goal setting and action planning were rare [14]. Moreover, patients should at least participate in setting and sharing these common “goals”, especially during the chronic phase of a condition. Even better would be goal-oriented care that encourages each patient to achieve the highest possible level of health as defined by the patient [15].

An important aspect of care coordination is collaboration between healthcare professionals. According to a study generating indicators of multidisciplinary teamwork, there is little multidisciplinary teamwork in primary care regarding the five most relevant indicators. There is a lack of culture, team relations, team leadership, team vision, and primary healthcare professionals perceived coordination of care [16]. Four of these indicators relate to key concepts of care coordination; these are “cultural factors, “quality of relationships”, “goals”, and “team outcomes”. Team leadership doesn’t relate to one of the identified key concepts of care coordination. Barriers and facilitators to working collaboratively were identified in an integrative review relating all to one of our identified key concepts, with the exception of reciprocity, hierarchical structures, and control. There were no factors identified relating to “patient characteristics” and “need for coordination” [17].

A previous study identified two theoretical frameworks for the study of care coordination as the most comprehensive including 11 of the 14 identified key concepts. These frameworks included the relational coordination theory and the multi-level framework [7]. The relational coordination theory states that organizational mechanisms influence the relational coordination, leading to a certain outcome [18,19]. The multi-level framework expands this theory and emphasizes that using the same organizational mechanisms both within and between organizations further strengthened relational coordination, resulting in greater quality and efficiency of care [20]. These frameworks should be expanded with “external factors”, “patient characteristics”, “cultural factors”, “quality of relationship” with patient, “team outcome”, and the links found in healthcare professionals’ experiences of care coordination.

Further research is required to evaluate the extent to which this theoretical framework helps with developing and assessing effective coordination strategies.

## Conclusion

This study explored key concepts of care coordination and links between these concepts from healthcare professionals’ experiences to develop a theoretical framework for the study of care coordination. The results show that external factors related to all (inter)organizational mechanism, which enhanced “roles”, “quality of relationship” between healthcare professionals and with the patient, “exchange of information”, and the setting and sharing of common “goals” to improve patient, team, and (inter)organizational outcomes. “Patient characteristics” was linked with “need for coordination” and “patient outcome”. We recommend that theoretical frameworks for the study of care coordination should be expanded with these interrelated concepts.
